# Fungal hyphae promote bacterial contact-dependent killing during surface-associated growth

**DOI:** 10.1093/ismejo/wraf135

**Published:** 2025-07-02

**Authors:** Miao Han, Chujin Ruan, Gang Wang, David R Johnson

**Affiliations:** College of Land Science and Technology, China Agricultural University, Beijing, China; Department of Environmental Microbiology, Swiss Federal Institute of Aquatic Science and Technology (Eawag), Dübendorf, Switzerland; Department of Environmental Microbiology, Swiss Federal Institute of Aquatic Science and Technology (Eawag), Dübendorf, Switzerland; College of Land Science and Technology, China Agricultural University, Beijing, China; Department of Environmental Microbiology, Swiss Federal Institute of Aquatic Science and Technology (Eawag), Dübendorf, Switzerland; Institute of Ecology and Evolution, University of Bern, Bern, Switzerland

**Keywords:** contact-dependent killing, antagonism, fungal hyphae, microbial interactions, spatial organization

## Abstract

Bacterial contact-dependent killing in spatially structured systems is shaped by physical constraints and biological interactions. In this study, we demonstrate the importance of fungal hyphae in facilitating bacterial dispersal and promoting contact-dependent killing during surface-associated growth. Using *Vibrio cholerae* as the killing bacterium and *Pseudomonas stutzeri* as the target bacterium, we show that fungal hyphae act as dispersal agents that facilitate bacterial spatial intermixing and promote contact-dependent killing. Specifically, we show that dispersal along fungal hyphae increases the number of contacts between *V. cholerae* and *P. stutzeri* cells, which in turn increases the extent of killing via the type VI secretion system encoded by *V. cholerae*. This enables *V. cholerae* to achieve growth dominance despite initial population disadvantages. We further show that the effect of fungal hyphae on the killing efficacy of *V. cholerae* depends on flagellar motility. Our study underscores the multifaceted effects of fungal hyphae in enhancing bacterial dispersal and intensifying interspecies interactions, highlighting the ecological significance of fungal–bacterial interactions in spatially structured systems.

## Introduction

Surface-associated bacterial systems, such as biofilms and colonies, are pervasive on our planet and have important roles in biogeochemical cycling, biotechnology, and human health and disease [1–3]. A typical characteristic of these systems is that they consist of densely packed cells, which can result in strong inter- and intraspecific competition for space and resources [4–6]. In the absence of bacterial dispersal pathways, the biomass expands across a surface as a consequence of cell shoving, which results in the decay of spatial intermixing between different bacteria along the biomass periphery [7, 8]. This is because only those cells located at the biomass periphery typically have access to nutrients replenished from the environment, which results in small effective population sizes that are subject to strong stochastic drift [7, 8]. Because many bacterial interactions occur at short ranges (i.e. at small length scales) [9], the decay in spatial intermixing and the corresponding increase in the mean distance between different bacteria can reduce the effectiveness of those interactions, with important consequences on resource utilization, ecosystem stability, and evolutionary trajectories [10–13].

Fungal hyphae and their associated thin water films can counteract the decay in spatial intermixing and reduce the mean distance between different bacteria during surface-associated growth [14]. Fungal hyphae are formed by many fungi and can traverse heterogeneous landscapes such as air–water interfaces [15–17]. Fungal hyphae also retain water films along their surfaces as a consequence of surface tension, creating dispersal pathways that enable bacteria to migrate away from local regions of high bacterial cell density where competition is fierce [18–22]. This increased dispersal can reduce the strength of competition between different bacteria, increase effective population sizes, and consequently counteract the effects of stochastic drift along the biomass periphery, thus slowing the decay in bacterial spatial intermixing during surface-associated growth [14, 23, 24].

Because fungal hyphae can increase spatial intermixing and reduce the mean distance between different bacterial cell types [14], we hypothesize that fungal hyphae can also strengthen short-range bacterial interactions. Indeed, hyphae-mediated increased numbers of cell contacts can promote horizontal gene transfer (HGT) [25] and facilitate bacterial foraging [26]. We therefore hypothesize that fungal hyphae can also increase the efficacy of contact-dependent killing, which is a prevalent antagonistic interaction that occurs between different bacteria. Contact-dependent killing requires direct contact between a killing cell and a target cell. If fungal hyphae increase spatial intermixing and reduce the mean distance between different bacteria [14, 20], then we expect more contacts between killing and target bacterial cells, consequently improving the killing efficacy. Of particular relevance is killing via the type VI secretion system (T6SS), which is a widespread mechanism among gram-negative bacteria [27, 28]. The T6SS is a nanomachine capable of delivering effector toxins directly into adjacent cells, providing a potent competitive advantage to killing cells [29–31]. However, an important constraint is that toxin delivery requires direct cell contact [32, 33].

Because toxin delivery requires direct cell contact, we posit that the increased spatial intermixing between killing and target bacterial cells caused by fungal hyphae will increase the killing efficacy. To test this hypothesis, we conducted surface-associated growth experiments with a T6SS-equipped strain of *Vibrio cholerae* as the killing bacterium [34] and *Pseudomonas stutzeri* as the target bacterium [11]. Although *V. cholerae* typically inhabits aquatic environments, it often grows within surface-associated systems, such as on phytoplankton, zooplankton, sediments, and various biotic and abiotic substrates [35, 36]. Its well-characterized T6SS positions *V. cholerae* as a model organism for investigating bacterial antagonism mediated by T6SS during surface-associated growth [31, 34, 37]. Notably, genomic surveys indicate that the T6SS is ubiquitous among fungal-associated bacteria [38], and the expression of T6SS genes can be upregulated during bacterial–fungal interactions [39], suggesting a crucial role in cross-kingdom interactions. However, in surface-associated systems such as in soil or marine biofilms, disentangling the relative contributions of physical dispersal along hyphae and biochemical interactions poses significant challenges. Thus, we employed a simplified and genetically tractable surface-associated system to precisely control and quantify how water films mediated by fungal hyphae modulate bacterial antagonism. We grew the strains together across nutrient-amended surfaces in the absence or presence of a hyphae-forming fungus. We then quantified the effects of fungal hyphae on bacterial spatial intermixing and competitive outcomes using confocal laser scanning microscopy (CLSM). Additionally, we quantified the role of bacterial motility on dispersal and spatial intermixing using a *V. cholerae* strain with a defect in flagellar motion [40]. By elucidating how fungal hyphae alter dispersal capabilities, bacterial spatial intermixing, and contact-dependent killing, we aimed to gain a deeper understanding of microbial community ecology and the interplay between the physical and biological drivers of short-range bacterial interactions.

## Results

### Fungal hyphae increase the spatial intermixing of *V. cholerae* and *P. stutzeri*

We hypothesized that fungal hyphae promote the spatial intermixing of *V. cholerae* and *P. stutzeri* by facilitating dispersal along their hyphal networks. To test this, we used a mutant of *V. cholerae* that has loss-of-function mutations in the *Hcp1* and *Hcp2* genes, which encode for essential structural components of the T6SS (referred to as *V. cholerae* ΔT6SS) [41]. This allowed us to isolate the effects of fungal hyphae from the effects of contact-dependent killing on the spatial intermixing of the different bacteria. To distinguish the strains, *V. cholerae* ΔT6SS expressed a green fluorescent protein-encoding gene [41], while *P. stutzeri* expressed a red fluorescent protein-encoding gene [42–44], both from the chromosome. To perform the experiment, we mixed *V. cholerae* ΔT6SS and *P. stutzeri* together in a 1:10 ratio (cell number:cell number) and grew them across nutrient-amended agar plates either in the presence or absence of the hyphae-forming fungus *Penicillium* sp. laika [14]. This fungus has no observable negative effects on the growth of *P. stutzeri* [14]. We first incubated the agar plates under oxic conditions at 21°C for 2 days to allow the fungus to form a dense hyphal network (Fig. S1). We then transferred the agar plates to anoxic conditions to inhibit further fungal growth and incubated them for an additional two days (Fig. S1). This allowed the bacteria to grow via denitrification and disperse on the hyphal network while preventing the passive movement of bacteria via further fungal growth (Fig. S1) [14]. We then imaged the biomass with CLSM and quantified the spatial intermixing and relative abundances of the bacteria from the images.

**Figure 1:**
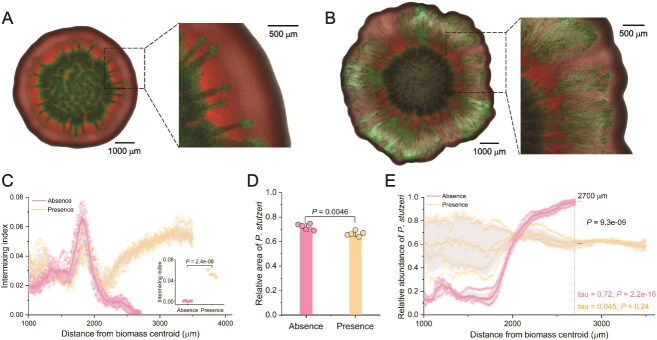
Surface-associated growth experiments with *V. cholerae* ΔT6SS and *P. stutzeri* in the absence or presence of fungal hyphae. Representative CLSM images (*n* = 5) after 4 days of growth in the (A) absence or (B) presence of fungal hyphae. *Vibrio cholerae* ΔT6SS expresses green fluorescent protein and *P. stutzeri* expresses red fluorescent protein. (C) The intermixing index as a function of the radial distance from the biomass centroid in the absence or presence of fungal hyphae (*n* = 5). The inset depicts the intermixing index at the biomass periphery (~2700 μm in the absence of fungal hyphae and ~ 3500 μm in the presence of fungal hyphae) (*n* = 5). (D) The relative biomass area of *P. stutzeri* after 4 days of growth in the absence or presence of fungal hyphae (*n* = 5). (E) The relative abundance of *P. stutzeri* as a function of the radial distance from the biomass centroid (*n* = 5). For (E), the colored tau and *P* value at the bottom of the panel are for Mann–Kendall trend tests. All other *P* values are for two-sample two-sided Welch tests.

**Figure 2:**
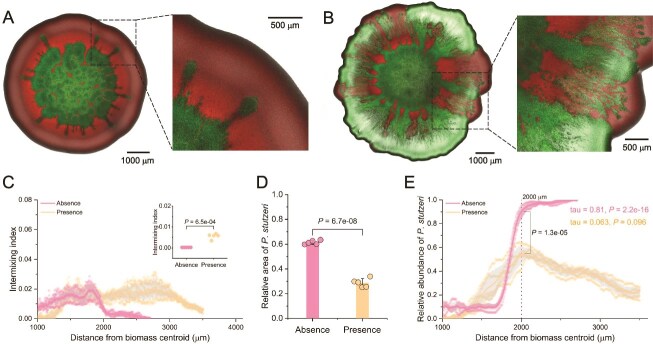
Surface-associated growth experiments with *V. cholerae* T6SS^+^ and *P. stutzeri* in the absence or presence of fungal hyphae. Representative CLSM images (*n* = 5) after 4 days of growth in the (A) absence or (B) presence of fungal hyphae. *Vibrio cholerae* T6SS^+^ expresses green fluorescent protein and *P. stutzeri* expresses red fluorescent protein. (C) the intermixing index as a function of the radial distance from the biomass centroid in the absence or presence of fungal hyphae (*n* = 5). The inset depicts the intermixing index at the biomass periphery (at ~2700 μm in the absence of fungal hyphae and ~ 3500 μm in the presence of fungal hyphae) (*n* = 5). (D) the relative biomass area of *P. stutzeri* after 4 days of growth in the absence or presence of fungal hyphae (*n* = 5). (E) The relative abundance of *P. stutzeri* as a function of the radial distance from the biomass centroid (*n* = 5). For (E), the colored tau and *P* values at the top of the panel are for Mann–Kendall trend tests. All other *P* values are for two-sample two-sided Welch tests.

We found that fungal hyphae did indeed substantially increase the spatial intermixing of *V. cholerae* ΔT6SS and *P. stutzeri* (Fig. 1A–C), which is consistent with previous studies [14]. This is particularly evident at the biomass periphery where growth is most vigorous (two-sample two-sided Welch test; *P* = 2.4 × 10^−8^, *n* = 5) (Fig. 2C). We also found that the presence of fungal hyphae marginally decreased the competitive ability of *P. stutzeri*, reducing its relative biomass area by 6% ± 1% when cocultured with *V. cholerae* ΔT6SS even though *V. cholerae* ΔT6SS did not express a functional T6SS (two-sample two-sided Welch test; *P* = .0046, *n* = 5) (Fig. 1D). This was associated with a reduction in the relative abundance of *P. stutzeri* at the biomass periphery from 96% ± 2% without hyphae to 61% ± 3% with hyphae (two-sample two-sided Welch test at a radial distance of 2700 μm; *P* = 9.3 × 10^−9^, *n* = 5) (Fig. 1E). When fungal hyphae were absent from the system, the relative abundance of *P. stutzeri* rapidly increased as the biomass expanded (Mann–Kendall trend test; tau = 0.72, *P* = 2.2 × 10^−16^) (Fig. 1E). However, when fungal hyphae were present, this effect was reduced and the relative abundance of *P. stutzeri* maintained a more consistent value (Mann–Kendall trend test; tau = 0.045, *P* = .24) (Fig. 1E).

### T6SS increases the competitiveness of *V. cholerae* when fungal hyphae are present

We tested whether T6SS-dependent killing enhances the competitive advantage of *V. cholerae* when grown together with *P. stutzeri*. We hypothesized that T6SS-dependent killing provides *V. cholerae* with a competitive advantage over *P. stutzeri* and that this advantage is enhanced in the presence of fungal hyphae. To test this, we performed the same experiments as described above, except we used the ancestral version of *V. cholerae* ΔT6SS that contains intact *Hcp1* and *Hcp2* genes (referred to as *V. cholerae* T6SS^+^) [34]. As we observed for *V. cholerae* ΔT6SS and *P. stutzeri*, we found that fungal hyphae substantially increased the spatial intermixing of *V. cholerae* T6SS^+^ and *P. stutzeri* (Fig. 2A–C). This is again most pronounced at longer radial distances from the biomass centroid, where the presence of fungal hyphae slowed the decay in spatial intermixing between the different bacteria (Fig. 2C). Furthermore, the spatial intermixing at the biomass periphery was significantly greater in the presence of fungal hyphae (two-sample two-sided Welch test; *P* = 6.5 × 10^−4^, *n* = 5) (Fig. 2C). Moreover, we again found that fungal hyphae significantly decreased the competitive ability of *P. stutzeri*, where the relative biomass area occupied by *P. stutzeri* was significantly smaller when fungal hyphae were present (two-sample two-sided Welch test; *P* = 6.7 × 10^−8^, *n* = 5) (Fig. 2D). This was again associated with a reduction in the relative abundance of *P. stutzeri* at the biomass periphery from 90% ± 4% in the absence to 54% ± 8% in the presence of fungal hyphae (two-sample two-sided Welch test at a radial distance of 2000 μm; *P* = 1.3 × 10^−5^, *n* = 5) (Fig. 2E). When fungal hyphae were absent, the relative abundance of *P. stutzeri* rapidly increased as the biomass grew (Mann–Kendall trend test; tau = 0.81, *P* = 2.2 × 10^−16^) (Fig. 2E). However, when fungal hyphae were present, this effect was eliminated and the relative abundance of *P. stutzeri* declined at radial distances > 2000 μm (Mann–Kendall trend test; tau = 0.063, *P* = .096) (Fig. 2E).

We found that the T6SS increased the competitiveness of *V. cholerae* when grown together with *P. stutzeri*. While fungal hyphae reduced the competitive ability of *P. stutzeri* regardless of whether the T6SS of *V. cholerae* was functional or not, the magnitude of the effect was larger when the T6SS was functional (Figs 1D and 2D). When the T6SS was inactivated (i.e. when we mixed *V. cholerae* ΔT6SS with *P. stutzeri*), fungal hyphae marginally reduced the relative biomass area of *P. stutzeri* by 6% ± 1% (72% ± 3% in the absence and 66% ± 2% in the presence) (Fig. 1D). In contrast, when the T6SS was active (i.e. when we mixed *V. cholerae* T6SS^+^ with *P. stutzeri*), fungal hyphae reduced the relative biomass area of *P. stutzeri* by 32% ± 2% (61% ± 2% in the absence and 29% ± 4% in the presence) (Fig. 2D). This difference in effect size was statistically significant (two-sample two-sided Welch test; *P* = 5.1 × 10^−9^, *n* = 5). Moreover, the relative abundance of *P. stutzeri* was generally lower when grown with *V. cholerae* T6SS^+^ than with *V. cholerae* ΔT6SS along the entire radial trajectory from the biomass centroid to the periphery, and this effect increased in size with radial distance from the biomass centroid (two-sample two-sided Welch test; *P* = .013 at a radial distance of 2000 μm, *P* = 5.5 × 10^−5^ at 3000 μm, *n* = 5) (Fig. S2). Thus, the initial reduction in *P. stutzeri* at the biomass periphery arises from the enhanced dispersal advantage of *V. cholerae* along the hyphal network, allowing it to reach and occupy the biomass periphery. When the T6SS is functional, this effect is further amplified because increased intermixing along hyphae promotes frequent cell–cell contacts and enhances contact-dependent killing.

We observed small finger-like protrusions formed by *V. cholerae* ΔT6SS or *V. cholerae* T6SS^+^ into the surrounding *P. stutzeri* biomass (Figs 1A and 2A). Because these strains are not known to secrete diffusible toxins that could induce bacterial antagonism, we expect the formation of these protrusions is likely a consequence of morphological differences between the strains, where the curved-rod shape of *V. cholerae* cells could enable them to rotationally wedge themselves into and physically invade the denser matrix of cylindrical-shaped *P. stutzeri* cells.

### Flagellar motility affects competitive outcomes when fungal hyphae are present

**Figure 3:**
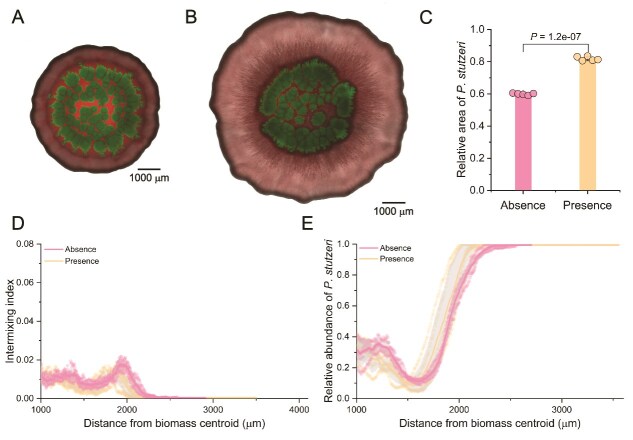
Surface-associated growth experiments with *V. cholerae* T6SS^+^Δ*flaA* and *P. stutzeri* in the absence or presence of fungal hyphae. Representative CLSM images (*n* = 5) after 4 days of growth in the (A) absence or (B) presence of fungal hyphae. *Vibrio cholerae* T6SS^+^Δ*flaA* expresses green fluorescent protein and *P. stutzeri* expresses red fluorescent protein. (C) The relative biomass area of *P. stutzeri* after 4 days of growth in the absence or presence of fungal hyphae (*n* = 5). The *P* value is for a two-sample two-sided Welch test. (D) The intermixing index as a function of the radial distance from the biomass centroid in the absence or presence of fungal hyphae (*n* = 5). (E) The relative abundance of *P. stutzeri* as a function of the radial distance from the biomass centroid (*n* = 5).

Although our data demonstrate that fungal hyphae affect competitive outcomes by increasing the efficacy of T6SS-mediated killing, we nevertheless observed a minor but significant effect of fungal hyphae when the T6SS was inactive, where the relative biomass of *P. stutzeri* reduced by ~6% (Fig. 1D). This minor effect indicates that hyphae can promote *V. cholerae*’s advantage even without contact-dependent killing. To explain how fungal hyphae could affect competitive outcomes other than by increasing the number of contacts between *V. cholerae* and *P. stutzeri* cells and the efficacy of T6SS-mediated killing, we posited that fungal hyphae might enable *V. cholerae* to disperse more effectively than *P. stutzeri* into locations where competition is weaker and resources are more abundant. To test this, we performed the same experiments as described above except that we used a version of *V. cholerae* T6SS^+^ that is deficient in flagellum assembly (referred to as *V. cholerae* T6SS^+^Δ*flaA* [40]) (Fig. 3A and B). We found that fungal hyphae had the opposite effect on *V. cholerae* T6SS^+^Δ*flaA* compared to what we observed with *V. cholerae* ΔT6SS and *V. cholerae* T6SS^+^. Specifically, the presence of fungal hyphae significantly increased the competitive ability of *P. stutzeri* when grown with *V. cholerae* T6SS^+^Δ*flaA*, with the relative biomass area of *P. stutzeri* increasing from 60% ± 1% in the absence to 82% ± 1% in the presence fungal hyphae (two-sample two-sided Welch test; *P* = 1.2 × 10^−7^, *n* = 5) (Fig. 3C). Indeed, *P. stutzeri* completely displaced *V. cholerae* T6SS^+^Δ*flaA* along the biomass periphery at radial distances from the biomass centroid >2000 μm (Fig. 3D and E). We observed no flagella-independent dispersal of *V. cholerae* T6SS^+^Δ*flaA*, confirming that loss of motility fully abrogates migration along hyphae under these conditions. Thus, even though *V. cholerae* had an intact T6SS, flagellar motility was nevertheless essential for *V. cholerae* to migrate into locations where resources were abundant and to remain competitive against *P. stutzeri.*

We validated that flagellar motility allows the bacteria to migrate to regions where competition is reduced and nutrients are more abundant, thus providing a growth benefit. To achieve this, we performed additional experiments as described above except that we grew each bacterium alone either with or without fungal hyphae (Fig. S3). We found that *V. cholerae* ΔT6SS, *V. cholerae* T6SS^+^, and *P. stutzeri* A1601 all achieved larger biomass areas when fungal hyphae were present (two-sided two-sample Welch tests; *P* < 10^−6^, *n* = 5) (Fig. S3A, B, and D). In contrast, *V. cholerae* T6SS^+^Δ*flaA* achieved a smaller biomass area when fungal hyphae were present (two-sided two-sample Welch test; *P* = .013, *n* = 5) (Fig. S3C). This effect remained at lower agar concentrations that activate swarming motility (Fig. S4). Thus, flagella are important for enabling the bacteria to migrate across fungal hyphae to locations where resources are more abundant.

We performed a third experiment to demonstrate the importance of flagellum-mediated motility where we collided colonies of bacteria with the fungi (Fig. S5). Briefly, we inoculated the fungi 5 mm apart from a mixture of *V. cholerae* T6SS^+^ and *P. stutzeri* or a mixture of *V. cholerae* T6SS^+^Δ*flaA* and *P. stutzeri* on nutrient-amended agar plates (Fig. S5A). In this experiment, only one side of the bacterial biomass had access to fungal hyphae for dispersal (Fig. S5B). As expected, upon contact with fungal hyphae, *V. cholerae* T6SS^+^ rapidly migrated to the biomass periphery and displaced *P. stutzeri* (Fig. S5C). In contrast, *V. cholerae* T6SS^+^Δ*flaA* was unable to migrate and was completely displaced by *P. stutzeri* at the bacterial–fungal interface (Fig. S5D). These findings confirm that a functional flagellum is essential for the migration of *V. cholerae* along fungal hyphae to locations where competition is reduced and nutrients are more abundant, thus determining competitive outcomes with *P. stutzeri*.

### Viable fungal hyphae are not necessary to explain the outcomes

We sought to test whether the two mechanisms governing competitive outcomes between *V. cholerae* and *P. stutzeri* described above (i.e. T6SS-mediated killing and flagellar-mediated dispersal) remain valid in the absence of viable fungal hyphae. *Penicillium* sp. laika stops growing under anoxic conditions but can resume growing when switched back to oxic conditions, and therefore maintains some metabolic activity [45, 46]. To remove any potential biological confounding factors of remaining metabolic activity, we performed experiments in which we replaced fungal hyphae with a glass filament, where the glass filament creates water films that mimic those produced by fungal hyphae. We expected that when bacteria encountered the glass filament, they would activate flagellar motion and disperse along the filament to locations where nutrients were more abundant. Simultaneously, they would increase spatial intermixing due to enhanced dispersal and promote T6SS-mediated killing. We performed these experiments by mixing *V. cholerae* ΔT6SS, *V. cholerae* T6SS^+^, or *V. cholerae* T6SS^+^Δ*flaA* with *P. stutzeri*, inoculating the mixtures onto nutrient-amended agar plates, and then placing a glass filament so that it touched the edge of the inoculation drop after it had evaporated completely.

**Figure 4:**
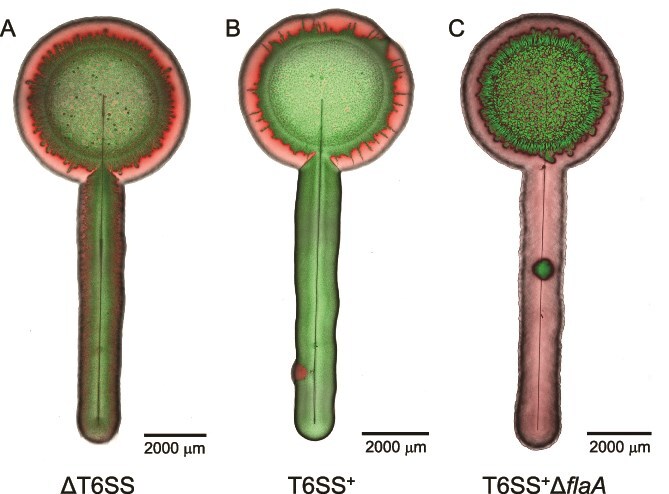
Surface-associated growth experiments along a glass filament as an abiotic surrogate for fungal hyphae. The images are representative CLSM images of *P. stutzeri* grown with (A) *V. cholerae* ΔT6SS, (B) *V. cholerae* T6SS^+^, or (C) *V. cholerae* T6SS^+^Δ*flaA*. The *V. cholerae* strains express green fluorescent protein and *P. stutzeri* expresses red fluorescent protein.

We found that both mechanisms (i.e. T6SS-mediated killing and flagellar-mediated dispersal) are indeed important even in the absence of viable fungal hyphae. When we grew *V. cholerae* ΔT6SS with *P. stutzeri*, we found extensive spatial intermixing of the bacteria along the glass filament (Fig. 4A). When we grew *V. cholerae* T6SS^+^ with *P. stutzeri*, we found that *V. cholerae* T6SS^+^ completely displaced *P. stutzeri* (Fig. 4B), presumably because there was sufficient spatial intermixing to foster a high efficacy of T6SS-mediated killing. Finally, when we grew *V. cholerae* T6SS^+^Δ*flaA* with *P. stutzeri,* we found that *P. stutzeri* completely displaced *V. cholerae* T6SS^+^Δ*flaA*, even though *V. cholerae* expressed a functional T6SS (Fig. 4C). This is because *P. stutzeri* was able to disperse more rapidly than *V. cholerae* along the glass filament to locations where nutrients were more accessible. We also observed sporadic discrete patches of the nondominant strain along the filaments (red in Fig. 4B; green in Fig. 4C). These patches occurred unpredictably across replicates and are unlikely to be imaging artifacts, possibly reflecting transient protective clustering or hitchhiking of nonmotile cells along the water film. We further verified the importance of flagellar motility by growing each bacterium alone along the glass filament (Fig. S6). Indeed, all the bacteria could rapidly migrate along the glass filament except *V. cholerae* T6SS^+^Δ*flaA*.

## Discussion

**Figure 5:**
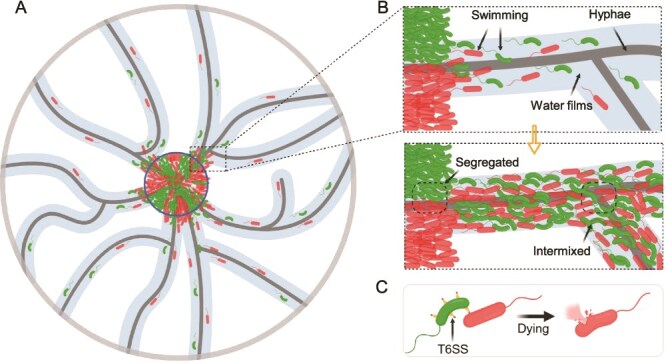
Schematic of how fungal hyphae promote bacterial contact-dependent killing. (A) The water films created by fungal hyphae can improve bacterial dispersal and consequently increase the spatial intermixing of different bacteria. (B) Bacterial dispersal along fungal hyphae is driven by flagella-mediated motility, which increases the spatial intermixing of different bacteria. (C) Increased bacterial dispersal will reduce the mean distance between killing and target bacterial cells, thereby enhancing the efficacy of contact-dependent killing.

Our study demonstrates that fungal hyphal networks can strengthen bacterial antagonistic interactions by modulating spatial organization during surface-associated growth (Fig. 5). By acting as dispersal agents via their associated water films [21, 22, 47], fungal hyphae increase the spatial intermixing of different bacterial strains (Fig. 5B), which reduces the mean distance between cells and increases the efficacy of contact-dependent killing (Fig. 5C). These findings provide mechanistic insights into microbial community assembly and highlight the ecological significance of fungal networks in shaping interspecies interactions and competitive outcomes [15, 48–51].

The presence of fungal hyphae significantly increases the spatial intermixing of *V. cholerae* T6SS^+^ and *P. stutzeri*, promotes the efficacy of T6SS-mediated killing, and ultimately confers a competitive advantage to *V. cholerae* T6SS^+^ over *P. stutzeri* (Fig. 2D). This is true even when the initial population ratio favors *P. stutzeri* (Fig. 2D). We found that fungal hyphae slow the decay in spatial intermixing at the biomass periphery caused by stochastic drift, which increases the number of contacts between killing (*V. cholerae*) and target (*P. stutzeri*) cells (Fig. 2C). This spatial proximity is critical for the T6SS mechanism, which relies on direct cell–cell contact to deliver toxins [30, 52–54]. In the absence of fungal hyphae, the rapid decay in spatial intermixing limits such interactions, reducing the efficacy of T6SS-mediated killing. This is evident from our experiments with *V. cholerae* ΔT6SS, which cannot dominate under these conditions (Fig. 1D). To disentangle the relative contributions of fungal hyphae and T6SS to competitive suppression, we quantified the reduction of *P. stutzeri* biomass for four experimental conditions (with or without fungal hyphae and with or without a functional T6SS). While each factor alone had a modest effect (fungal hyphae: 6%; T6SS: 11%), their combination led to a pronounced 37% reduction (Figs 1D and 2D). The antagonistic impact of T6SS was strongly amplified in the presence of hyphae. These findings underscore a synergistic interaction, where fungal-hyphae-facilitated intermixing substantially boosts the efficacy of contact-dependent killing, identifying T6SS as the dominant yet context-dependent driver of competitive exclusion in the mycosphere.

Experiments with glass filaments further demonstrate that viable fungal hyphae are not necessary for the two mechanisms to manifest (i.e. T6SS-mediated killing and migration to locations with abundant resources). Even under anoxic conditions, biochemical interactions between fungi and bacteria cannot be ignored. For example, chitin in fungal cell walls can induce T6SS expression in *V. cholerae* via ChiS activation [55]. However, our parallel experiments using glass filaments generated similar outcomes on bacterial dispersal, spatial intermixing, and T6SS-mediated killing, indicating that the critical factor is the formation of water films created by hyphal networks rather than chitin-mediated signaling. These findings highlight how fungal hyphae mitigate spatial constraints to enhance T6SS functionality, providing antagonistic strains with a competitive advantage and emphasizing the close link between T6SS killing efficacy and spatial organization [14, 56].

Fungal hyphae affect competitive outcomes between killing and target cells not only by modulating spatial organization but also by enabling flagellum-mediated motility. Previous studies have shown that the efficacy of flagella-driven dispersal is influenced by the physicochemical surface properties of the hyphae, including hydrophobicity and composition of the extracellular matrix [21, 22]. We therefore acknowledge that the relative advantage conferred by hyphal networks can vary depending on fungal species or environmental contexts, which may alter the continuity and thickness of the water film essential for bacterial motility. In our case, the competitive success of *V. cholerae* ΔT6SS is increased in the presence of fungal hyphae even though this bacterium does not engage in killing (Fig. 1D). Importantly, this effect vanishes with *V. cholerae* T6SS+Δ*fla*, which can kill but not undergo flagellar motion (Fig. 3C)*.* This illustrates the multifaceted effects of fungal hyphae on competitive outcomes, where they can impact competition by two mechanisms: by modulating spatial organization to increase cell contacts and killing efficacy and by enabling cells to migrate to regions where competition is reduced and resources are more abundant [57, 58].

Our findings have broader implications for microbial ecology and evolution. By facilitating bacterial dispersal and promoting interspecies contacts, fungal hyphae likely influence the selection pressures acting on microbial systems [51, 59–61]. For instance, the enhanced efficacy of T6SS-mediated killing in hyphal networks could drive the evolution of competitive traits, such as toxin production, motility, and resistance mechanisms [62–66]. Conversely, fungal networks may foster cooperative behaviors, such as nutrient sharing or biofilm formation, depending on the environmental context [23, 67, 68]. These dual roles—as mediators of both competition and cooperation—position fungal networks as critical ecological hubs within microbial ecosystems. Fungal hyphal networks not only facilitate dispersal but also enable bacterial foraging along continuous water films, as demonstrated for the predation of *Bdellovibrio bacteriovorus* [26] and for chemotactic degraders of polycyclic aromatic hydrocarbons [22]. Fungal highways also serve as focal points for HGT, where plasmid conjugation occurs more readily along hyphal networks, suggesting that hyphae accelerate the spread of adaptive traits in spatially structured communities [14, 25]. Ubiquitous in natural environments, fungal hyphae interact with diverse microbial communities, contributing to nutrient cycling [15, 51, 69], soil structure [70], and plant–microbe interactions [58, 61]. Our results suggest that fungal hyphal networks also play a pivotal role in modulating microbial antagonism and community assembly, balancing positive and negative interactions in shaping microbial ecosystems.

The effects of fungal hyphal networks can be linked to metacommunity ecology, providing a novel perspective on the connectivity of spatially separated microbial communities [71, 72]. Within the framework of metacommunity ecology, fungal hyphae not only regulate local community dynamics but also enhance the complexity of species interactions across spatial scales through dispersal functions [15, 60, 73]. This cross-scale integration may influence community diversity, stability, and adaptability in natural environments [49, 74, 75]. Analogous dynamics can be observed in macroecological systems. For instance, in savanna ecosystems, apex predators such as lions rely on their mobility to traverse fragmented habitats, effectively linking prey populations across different local patches. Lions often use landscape features, such as riverbanks or dense vegetation, as corridors to access prey, similar to how fungal hyphal networks serve as conduits for bacterial dispersal [16]. However, key differences exist between the two systems. Fungal hyphae form a continuous growth network that also supplies nutrients to associated bacteria, unlike discrete landscape patches in savannas. In both systems, spatial connectivity is critical for predator–prey interactions [76–78]; while lions, like *V. cholerae*, require close contact with prey to exert their predatory pressure, their cooperation depends on pride composition and individual roles, whereas microbial cell density along hyphae can directly trigger contact-dependent antagonism via quorum sensing.

In conclusion, our study highlights the critical role of fungal hyphae in shaping bacterial competition by two mechanisms: slowing the decay in spatial intermixing at the biomass periphery, thus maintaining more cell contacts, and serving as dispersal conduits to facilitate flagellum-mediated motility. These synergistic effects amplify contact-dependent killing and enhance antagonistic interactions. These findings provide a mechanistic foundation for understanding how hyphae drive microbial community assembly and dynamics and underscore the ecological significance of fungal–bacterial interactions. By bridging spatial and functional aspects of microbial ecology, our work opens new avenues for exploring the complex interplay between fungi and bacteria in natural and engineered environments.

## Materials and methods

### Strains and growth conditions

We used *V. cholerae* strain 2740-80 as the parental strain, which is a streptomycin-resistant nontoxinogenic El Tor strain. *Vibrio cholerae* T6SS^+^ contains a deletion in *vipA* complemented by a carboxy-terminal *vipA–msfGFP* fusion inserted into the native *vipA* locus under control of the endogenous *vipA* promoter [34]. *Vibrio cholerae* ΔT6SS contains deletions in the *hcp1* and *hcp2* genes, which encode essential structural components of the T6SS [41]. *Vibrio cholerae* T6SS^+^Δ*flaA* (KDV956) is a derivative of *V. cholerae* strain C6706 (KDV201), which is known to be proficient in T6SS-mediated killing [66]. This strain contains a deletion in the *flaA* gene encoding the major flagellin subunit [40] and carries the plasmid pNUT542 expressing *msfGFP* under the isopropyl β-D-1-thiogalactopyranoside (IPTG)-inducible P*_tac_* promoter [79]. We used *P. stutzeri* A1601-mCherry as the target strain for all our experiments, which contains a loss-of-function deletion in the *comA* gene to prevent recombination, a chromosomally integrated gentamicin resistance gene for contamination control, and a chromosomally integrated IPTG-inducible P*_lac_* promoter located immediately upstream of the *echerry* gene [11, 43]. Our main outcomes are independent of promoter strengths, as all key comparisons are within each strain and yield consistent spatial and competitive patterns regardless of fluorescence expression level. We cultured all bacterial strains at 37°C in Luria–Bertani (LB) medium or on LB agar plates containing 1.5% agar and supplemented with appropriate antibiotics or IPTG. Prior to experiments, we grew each strain individually in overnight liquid cultures, centrifuged the cells at 1000 × *g* for 5 minutes, discarded the supernatants, and resuspended the cell pellets in fresh LB medium. We repeated the washing procedure three times. We then adjusted the cell densities based on their optical densities at 600 nm (OD_600_) to the desired levels in LB medium.

We used the hyphae-forming fungus *Penicillium* sp. laika for all our experiments [14]. We cultured the strain in liquid LB medium or on LB agar plates containing kanamycin (50 mg ml^−1^) to prevent bacterial contamination. This strain forms villiform colonies when grown on LB agar plates at 20°C. To prepare spores, we grew *Penicillium* sp. laika on LB agar plates for 5 days in oxic conditions. We then collected spores using a sterile loop, suspended the spores in 1 ml of 0.9% (*w*/*v*) NaCl, vortexed the suspension for 10 minutes to ensure dispersion, and adjusted the OD_600_ to 1 for subsequent experiments.

### Surface-associated growth experiments

We performed surface-associated growth experiments on LB agar plates supplemented with 5 mM sodium nitrate to support bacterial growth under anoxic conditions. We prepared the plates by autoclaving the medium at 121°C for 20 minutes, cooling to 70°C, adding 100 mM IPTG or appropriate antibiotics, and pouring 10 ml into 3.5-cm sterile Petri dishes. We then solidified the plates at room temperature, dried them under sterile conditions, sealed them with Parafilm (Amcor, Zürich, Switzerland), and stored them at 4°C until use.

For each experiment, we first grew overnight cultures of *V. cholerae* T6SS^+^, *V. cholerae* ΔT6SS, *V. cholerae* T6SS^+^Δ*flaA* or *P. stutzeri* A1601 alone in liquid LB medium, washed the cells with fresh LB medium as described above, and adjusted the OD_600_ to achieve 2 × 10^5^ (all *V. cholerae* strains) or 2 × 10^6^ (*P. stutzeri*) colony-forming units (CFU) ^—^per milliliter. We then mixed one volume of *V. cholerae* T6SS^+^, *V. cholerae* ΔT6SS, or *V. cholerae* T6SS^+^Δ*flaA* with one volume of *P. stutzeri* A1601. We next added an equal volume of 0.9% NaCl solution with or without *Penicillium* sp. laika spores to the bacterial mixtures. After mixing, we added 1-μl droplets of the mixture onto the centers of replicated LB agar plates (one drop per plate) and incubated the agar plates under oxic conditions at 21°C for 2 days to allow a fungal hyphal network to form (Fig. S1). We then transferred the plates to an anoxic glove box (Coy Laboratory Products, Grass Lake, MI) containing a 97% N₂/3% H₂ atmosphere and incubated the plates at 21°C for an additional 2 days (Fig. S1). This stopped fungal growth and prevented passive bacterial dispersal while allowing *P. stutzeri* to continue growing via denitrification. We included sodium nitrate in the medium to support anaerobic respiration via denitrification, particularly for *P. stutzeri*, which possesses a complete denitrification pathway [44]. *Vibrio cholerae*, although lacking nitrite reductase, can still reduce nitrate via NapA under neutral pH conditions to maintain viability, allowing it to survive under anoxic conditions [80]. After incubation, we removed all the plates from the glove box and exposed them to ambient air at 4°C for 2 hours to promote maturation of the fluorescent proteins. We also performed additional experiments as described above except that we grew each bacterial strain alone either with or without fungal hyphae. We performed all the experiments with five independent biological replicates.

For experiments conducted with glass filaments, we purchased filaments with diameters of ~48 μm and a length of 1 cm (P-D Glasseiden, Oschatz, Germany) [14, 25] and used them as abiotic surrogates of fungal hyphae. We next prepared and mixed the bacterial strains following the same protocol as above except that we adjusted the initial bacterial concentrations to 10^7^ CFU ml^−1^ for all *V. cholerae* strains and 10^8^ CFU ml^−1^ for *P. stutzeri* A1601. Specifically, we first spotted 1-μl droplets of the bacterial mixture onto the centers of separate LB agar plates (one drop per plate) and allowed the droplets to fully evaporate to prevent passive bacterial migration along water films during filament placement. Once dry, we carefully positioned a single sterile glass filament over the inoculation area for each agar plate. We then sealed the agar plates with Parafilm and incubated them under oxic conditions at 21°C for 5 days to allow active bacterial expansion along the glass filaments.

### Microscopy and image analysis

We acquired images of microbial biomass using a Leica TCS SP5 II confocal laser scanning microscope equipped with a 2.5× HCX FL objective (NA 0.12, 1024 × 1024 pixels, pixel size 6.055 μm). We excited green fluorescent protein at 488 nm and red fluorescent protein at 514 nm. We set the emission filters to 519–551 nm for green fluorescent protein and to 601–650 nm for red fluorescent protein. We collected images at the focal plane of strongest fluorescence signaling. We did not perform complete *z*-stacks as our prior work demonstrated little qualitative variance in the *z*-direction [11].

We performed quantitative image analyses using ImageJ 1.52i (https://imagej.net). To obtain the total relative area of *P. stutzeri* across the entire biomass area, we first applied a threshold to the intensity of each fluorescence channel and measured the fluorescent area limited to the threshold of each fluorescence channel. We calculated the total relative biomass area of *P. stutzeri* by dividing the red fluorescent area by the total fluorescent area. We measured the spatial intermixing of different bacteria using the “Sholl” plugin in ImageJ [81] using a well-established intermixing index [12, 14, 82]. Briefly, we first applied an autothreshold to one of the fluorescence channels using the Huang algorithm implemented in ImageJ. We then used the “Sholl” analysis plugin to calculate the number of intersections between the background and fluorescence signal at 5-μm increments from the centroid of the inoculation area to the periphery of the final biomass region. We quantified the intermixing index (*I_r_*) as


$$ {I}_r=\frac{N_r}{\pi r/2} $$

We calculated the relative abundance of *P. stutzeri* as a function of the radial distance from the biomass centroid. We first selected the biomass area using the “Wand (tracing)” tool and applied a threshold to the intensity of each fluorescence channel to remove noise. We then used the “Radial Profile” plugin to calculate the normalized fluorescence intensity as a function of the radial distance from the biomass centroid.

### Statistical analyses

We used the Welch *t*-test for all pairwise comparisons with IBM SPSS Statistics 24 (IBM Corp. Armonk, NY, USA), and we therefore did not make any assumptions regarding the homoscedasticity of our datasets. We used the Mann–Kendall trend test to test for relationships between spatial organization features and the radial distance from the biomass centroid (note that the radial distance from the biomass centroid is approximately equivalent to the incubation time in our study) with the “trend” package in the RStudio software (version 4.2.1, RStudio Team, PBC, Boston, MA, USA). We considered *P* < 0.05 to be statistically significant. We plotted all the graphics using Origin 2024b (OriginLab Corporation, Northampton, MA, USA).

## Supplementary Material

20250626_SI_Han_R2_wraf135

## Data Availability

All the data and code generated in this study have been deposited in the Eawag Research Data Institutional Repository (https://opendata.eawag.ch/) and are freely available to the public at doi:10.25678/000ECG. All the microbial strains are available from the corresponding authors upon request.
