# Supplementary material for: Fungal hyphae promote bacterial contact-dependent killing during surface-associated growth
Source: ISME J. 2025 Jul 2;19(1):wraf135. doi: 10.1093/ismejo/wraf135 (PMC12448445; doi:10.1093/ismejo/wraf135)
Supplement: 20250626_SI_Han_R2_wraf135 [file 20250626_si_han_r2_wraf135.pdf]

SUPPLEMENTARY INFORMATION FOR

Fungal hyphae promote bacterial contact-dependent killing during surface-associated growth

Miao Han[1, 2#], Chujin Ruan[2#], Gang Wang[1*], David R. Johnson[2, 3*]

[1]College of Land Science and Technology, China Agricultural University, Beijing, China; [2]Department of Environmental Microbiology, Swiss Federal Institute of Aquatic Science and Technology (Eawag), Dübendorf, Switzerland; [3]Institute of Ecology and Evolution, University of Bern, Bern, Switzerland. [#]These authors contributed equally: Miao Han, Chujin Ruan.

CORRESPONDENCE:
David R. Johnson: david.johnson@eawag.ch; Gang Wang: gangwang@cau.edu.cn

THIS FILE INCLUDES

Figs. S1-S6

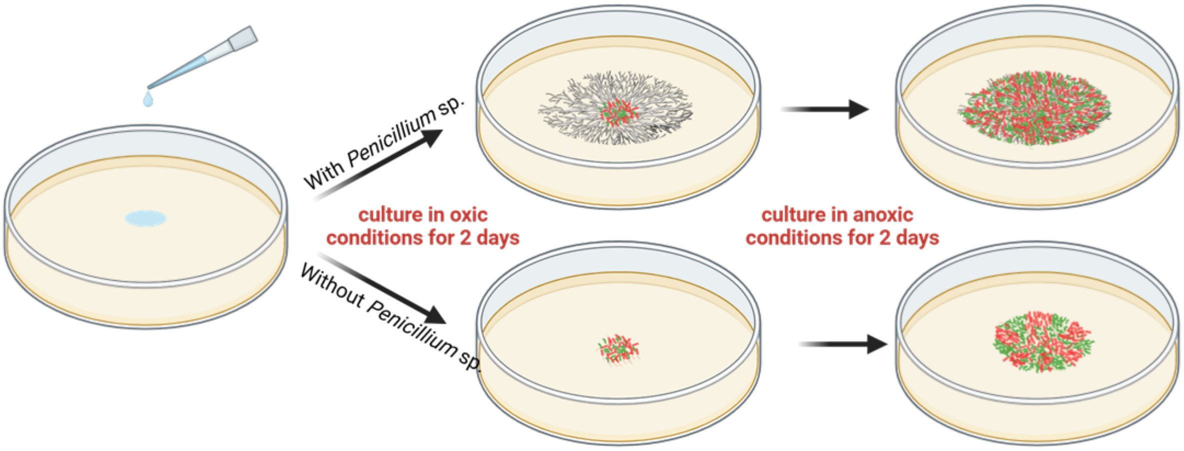

Fig. S1 Experimental system and approach. We mixed *Vibrio cholerae* as the killing bacterium and *Pseudomonas stutzeri* as the target bacterium with or without the hyphae-forming fungus *Penicillium* sp. laika and inoculated them onto nutrient-amended agar plates. We incubated the agar plates at 21°C in oxic conditions for two days, which enabled the formation of a dense hyphal network. We then transferred the agar plates to anoxic conditions for another two days, which enabled the bacterial strains to continue growing via denitrification and disperse along the hyphal network while preventing the fungus from further growing. In parallel, we performed control experiments in the absence of the fungus.

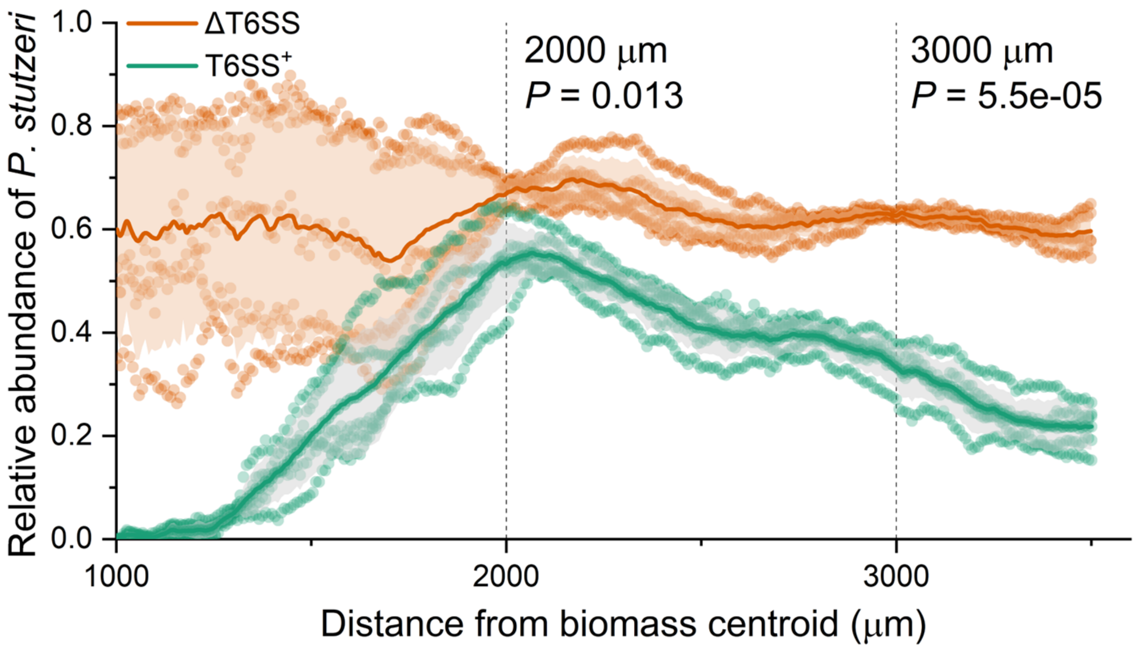

Fig. S2 Relative abundance of *P. stutzeri* when co-cultured with *V. cholerae* ΔT6SS or *V. cholerae* T6SS$^+$ in the presence of fungal hyphae as a function of distance from the biomass centroid. Relative abundance is the proportion of the biomass occupied by *P. stutzeri* at each radial distance from the biomass centroid. Data points are for five independent replicates at each radial distance, the solid lines are the average values, and the shaded regions are one standard deviation. The *P* values are for two-sample two-sided Welch tests at the indicated radial distances.

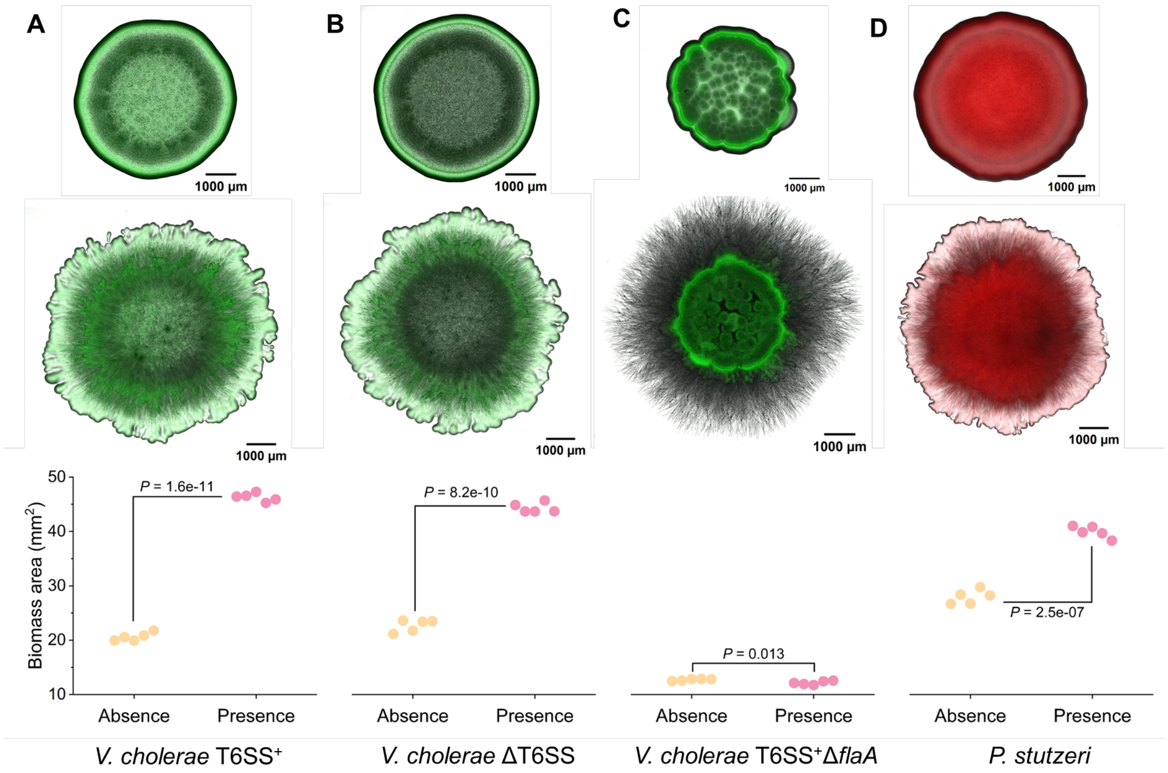

Fig. S3 Surface-associated growth experiments of individual bacteria in the absence or presence of fungal hyphae. The images are representative CLSM images of (**A**) *V. cholerae* T6SS$^+$, (**B**) *V. cholerae* ΔT6SS, (**C**) *V. cholerae* T6SS$^+$Δ*flaA*, or (**D**) *P. stutzeri* after four days of growth in the absence (top) or presence (middle) of fungal hyphae. The *V. cholerae* strains are green and *P. stutzeri* is red. The *P* values are for two-sample two-sided Welch tests.

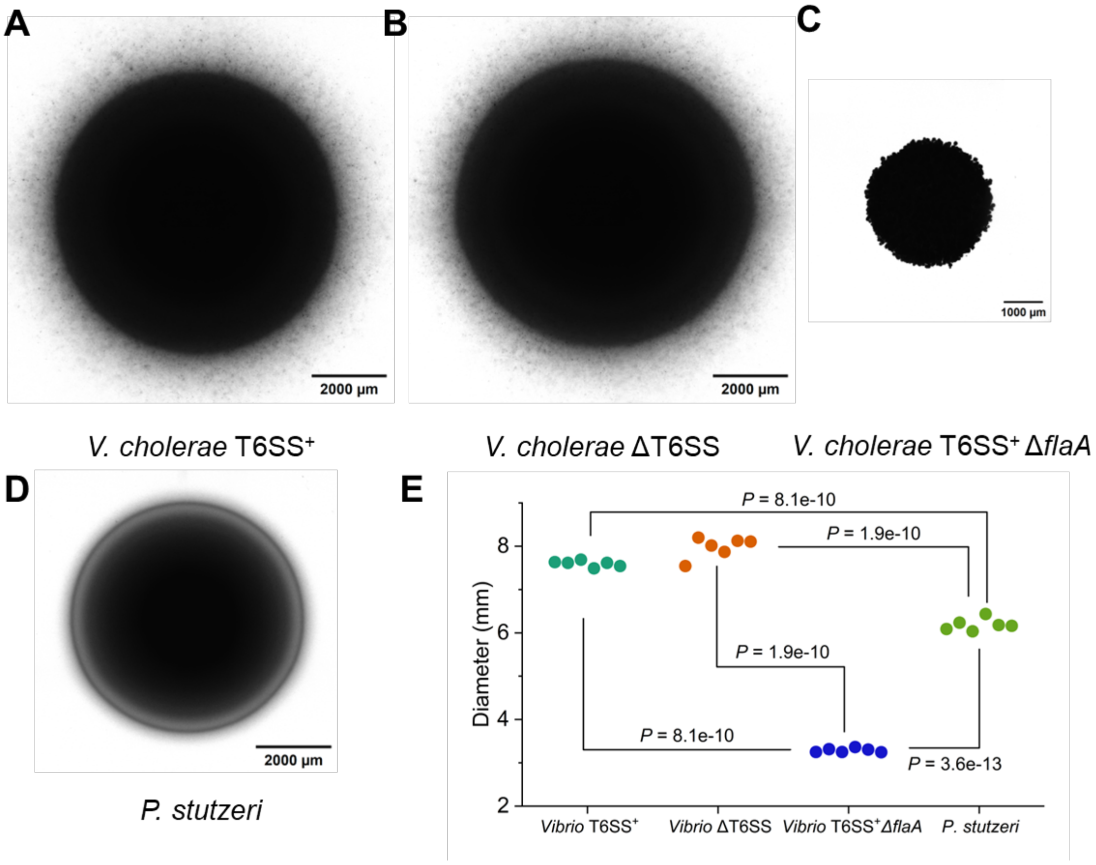

Fig. S4 Swarming behavior of individual bacteria. Images are representative microscopic images of (**A**) *V. cholerae* T6SS^{+}, (**B**) *V. cholerae* ΔT6SS, (**C**) *V. cholerae* T6SS^{+}Δ*flaA* or (**D**) *P. stutzeri* after 24 hours of growth on nutrient-amended agar plates containing 0.2% agar. The *P* values are for two-sample two-sided Welch tests.

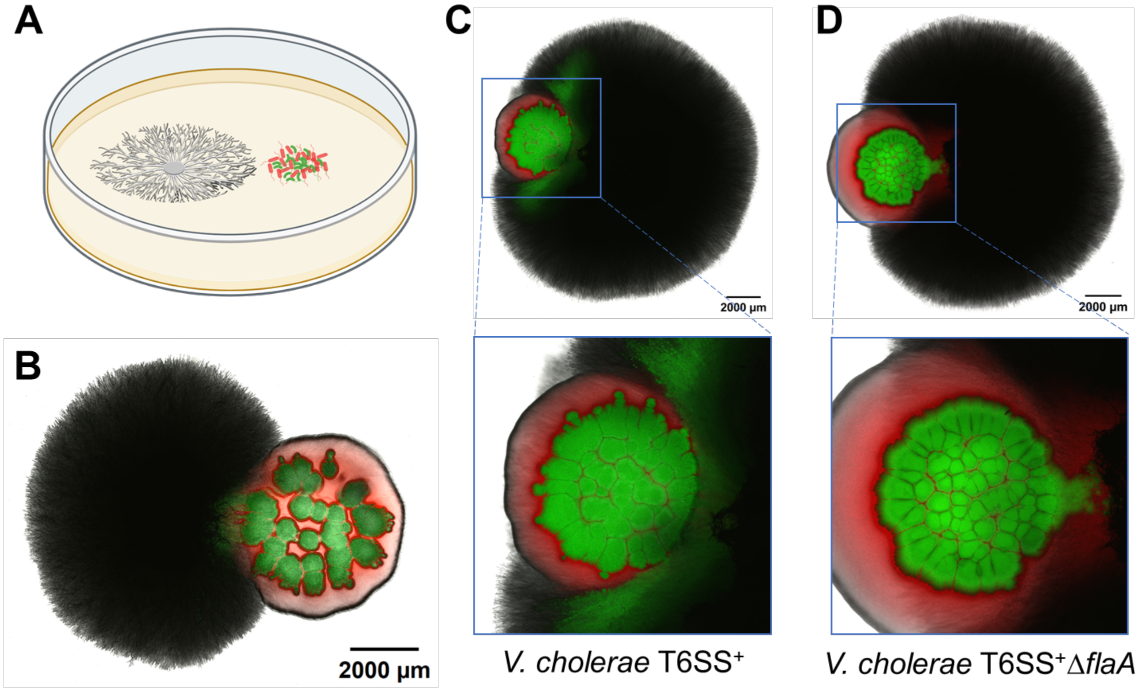

Fig. S5 Collision experiments between co-cultures of *P. stutzeri* and *V. cholerae* with *Penicillium* sp. laika. (**A**) We mixed *V. cholerae* T6SS^{+} or *V. cholerae* T6SS^{+}Δ*flaA* with *P. stutzeri* and inoculated the mixtures onto the surfaces of nutrient-amended agar plates while inoculating the fungal droplets at a distance of 5 mm from the centroid of the bacterial inoculation droplet. (**B**) Representative CLSM image (n = 5) as the bacterial biomass formed by *V. cholerae* T6SS^{+} with *P. stutzeri* initially comes into contact with the fungal biomass after three days of inoculation (**C** and **D**) Representative CLSM image (n = 5) after the biomass formed by (**C**) *V. cholerae* T6SS^{+} and *P. stutzeri*, or (**D**) *V. cholerae* T6SS^{+}Δ*flaA* and *P. stutzeri* came into contact with the fungal biomass after five days of growth. For all panels, the *V. cholerae* strains are green and *P. stutzeri* is red.

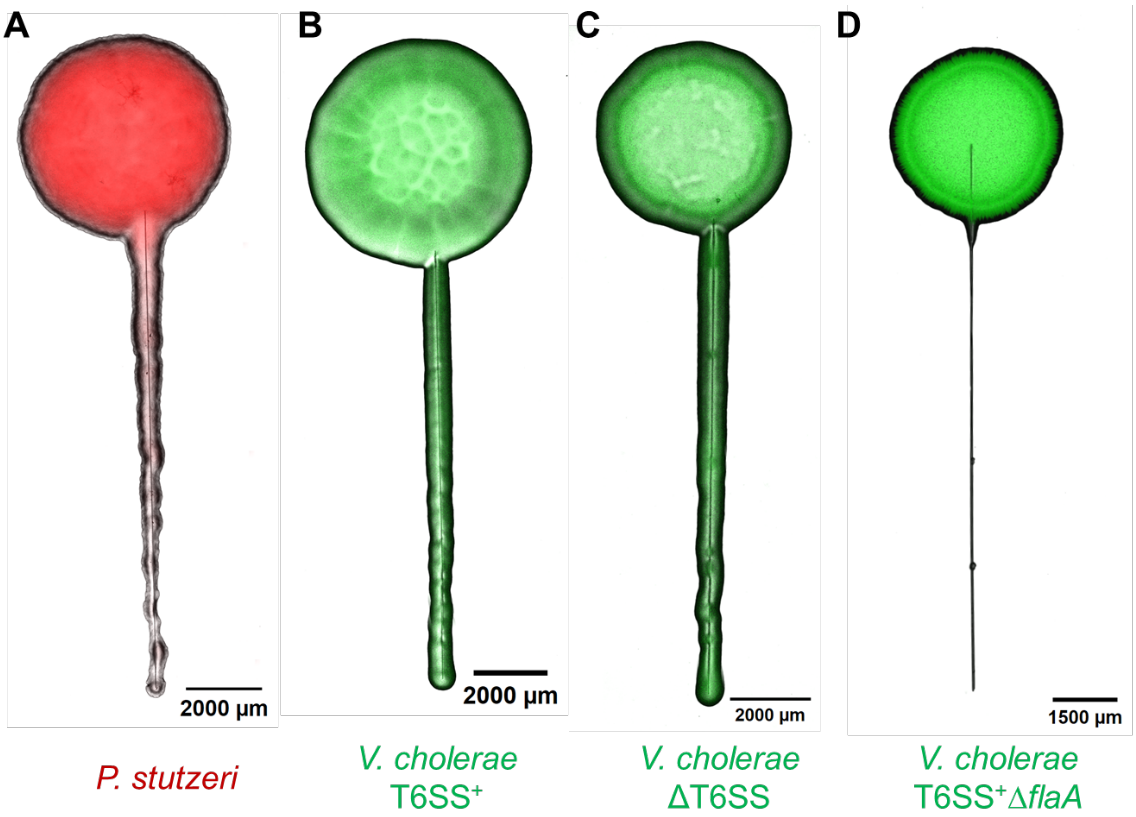

Fig. S6 Surface-associated growth experiments within individual bacteria along a glass filament as an abiotic surrogate for fungal hyphae. The images are representative CLSM images of (**A**) *P. stutzeri*, (**B**) *V. cholerae* T6SS^{+}, (**C**) *V. cholerae* ΔT6SS, or (**D**) *V. cholerae* T6SS^{+}Δ*flaA*. The *V. cholerae* strains are green and *P. stutzeri* is red.
